# Characteristics and efficacy of physical activity interventions to improve cardiometabolic and psychosocial outcomes in people living with HIV in sub-Saharan Africa: a protocol for a systematic review

**DOI:** 10.1186/s13643-023-02186-5

**Published:** 2023-02-23

**Authors:** SZ Mabweazara, J Manne-Goehler, M Hamer, J Cellini, MJ Siedner

**Affiliations:** 1 Africa Health Research Institute, Africa Centre Building, Route 618, Somkhele, KwaZulu Natal South Africa; 2 Massachusetts General Hospital, Boston, USA; 3 Harvard Medical School, Boston, USA; 4 University College London, London, UK

**Keywords:** HIV, Physical activity, Cardiometabolic, Sub-Saharan Africa, People living with HIV, Systematic review

## Abstract

**Background:**

Antiretroviral therapy (ART) has led to an increased lifespan for people living with HIV (PWH). This increased lifespan, coupled with the effects of HIV and adverse effects of ART have resulted in an increasing burden of cardiometabolic disease (CMD) among PWH. Physical activity (PA) has been proposed as an effective strategy to reduce the risk of developing cardiometabolic disease and other health complications in PWH. The aim of this paper is to review the characteristics and efficacy of PA interventions to improve cardiometabolic and psychosocial outcomes among PWH in sub-Saharan Africa.

**Methods:**

The review will follow the preferred reporting items for systematic review and meta-analysis protocols (PRISMA-P). Literature searches will be conducted in PubMed, Web of Science (WoS), African Index Medicus, Cumulative Index to Nursing and Allied Health Literature (CINAHL), and Embase. Peer-reviewed publications will be included if they include adults (age 18 or older), PWH in sub-Saharan Africa, and a PA intervention to improve cardiometabolic outcomes and/or psychosocial outcomes. We will include randomized controlled trials and quasi-experimental study designs. Two independent reviewers will screen all abstracts and full-text articles. The study methodological quality (or bias) will be appraised using the Revised tool to assess risk of bias in randomized trials and the Downs and Black checklist. Certainty of evidence will be evaluated using the Grading of Recommendations Assessment, Development and Evaluation guidelines. Meta-analyses will be conducted if our results are adequate for meta-analysis. Outcomes will be analyzed as continuous or dichotomous and meta-analyses will be conducted using random effects models with Stata computer software.

**Discussion:**

This review will identify and synthesize the current evidence regarding the characteristics and efficacy of PA interventions to improve cardiometabolic and psychosocial outcomes among PWH in sub-Saharan Africa. We also plan to identify the strengths and weaknesses of evaluated interventions. Based on the evidence, recommendations will be made to promote the design and further evaluate the most promising strategies to maximize the efficacy of PA interventions in improving cardiometabolic and psychosocial outcomes in PWH in sub-Saharan Africa.

**Systematic review registration:**

PROSPERO registration ID: CRD42021271937.

**Supplementary Information:**

The online version contains supplementary material available at 10.1186/s13643-023-02186-5.

## Background

Even though sub-Saharan Africa only makes up about 11% of the world population, it remains the global epicenter of the HIV epidemic. Of the 37 million global HIV infections, it is estimated that 20.6 million and 4.7 million HIV infections were found in Eastern and Southern Africa and Western and Central Africa respectively [1]. But notably, health priorities for people living with (PWH) have meaningfully changed in the region. The advent and global distribution of antiretroviral therapy (ART) is has led to a normalization of the lifespan of people living with HIV (PWH) [2]. Thus, ART has changed HIV from a nearly routinely fatal condition, to a manageable chronic disease that extends life expectancy to near that of uninfected people when initiated early and maintained [3, 4].

The widespread use of ART and increasing life expectancy for PWH has concomitantly led to an increased burden of non-communicable diseases among this population [5]. Cardiovascular and metabolic diseases (CMDs) have been found to be the most prevalent and most morbid non-infectious among PWH [6]. It is thus not surprising that, even though there is a narrowing life expectancy gap between PWH and people without HIV, PWH live fewer comorbidity-free years than people without HIV, and this is not improving over time [4]. This finding is also supported by a study with more than 37,000 adult PWH who were matched with up to three HIV-negative control subjects, which compared the rates of co-morbid conditions and trends over time among PWH and HIV-negative people [7]. The study found that PWH had higher comorbidity rates than HIV-negative people and that the greater rate of comorbidities, which included CMDs, osteoporosis and fractures, hyperlipidemia and endocrine disease increased with time among PWH [7]. Among these comorbidities, hypertension has been found to be the leading risk factor for cardiovascular disease in PWH in sub-Saharan Africa [8]. A higher risk for diabetes mellitus has also been reported for PWH on ART compared to people without HIV [9, 10]. PWH are also becoming more obese with rates ranging from 5% in Nigeria [11] to 23% in South Africa [12]. Recently, The Randomized Trial to Prevent Vascular Events in HIV (REPRIEVE) reported poor cardiovascular health among PWH as measured by Life’s Simple 7 (LS7), which includes smoking, diet, physical activity (PA), body mass index (BMI), blood pressure, total cholesterol, and glucose [13]. Poor dietary and PA patterns were reported on LS7 among the participants [13]. Specifically, for PA, out of a total of 7382 participants, only 773(11%) had an ideal PA score on LS7 and 2949 (40%) had poor PA scores on LS7 [13].

In summary, this body of literature suggest that with use of lifelong ART, PWH approach a normal life expectancy, and that priorities for health among PWH are shifting from preventing opportunistic infections to maintaining a healthy life and preventing/managing CMDs. Thus, more attention to comorbidity prevention for PWH is warranted and the critical and independent role for lifestyle interventions together with conventional treatment to improve cardiometabolic health outcomes in PWH must be prioritized. This is particularly important since ART has been found to be associated with unwanted adverse effects [14], including inducing cardiometabolic toxicities [15], which may eventually lead to CMD. The Action in Diabetes and Vascular Disease-PreterAx and DiamicroN Controlled Evaluation (ADVANCE) and the New Antiretroviral and Monitoring Strategies in HIV-infected Adults in Low-Income Countries (NAMSAL) studies, two large clinical trials in sub-Saharan Africa, found that women on dolutegravir-based ART treatment have a significantly higher risk of becoming overweight or obese [16]. In response, interventions which reduce the risk of obesity and its CMD downstream effects are urgently needed in this population.

PA has been shown to be an effective strategy to reduce the risk of developing CMD in PWH [17] and to effectively address the impairments caused by the virus itself and ART [18]. For example, resistance exercise is associated with significant decreases in total cholesterol, triglycerides, and low-density lipoprotein and increased high-density lipoprotein [19]. A resistance exercise intervention among PWH with metabolic syndrome led to significant decreases in fasting glucose, haemoglobin A1C (HbA1c) and body fat percentage compared to controls [20]. After the intervention, those in the exercise arm of the intervention no longer had metabolic syndrome [20]. A second study investigating blood pressure responses during and after one bout of resistance exercise in women with HIV found clinically relevant decreases (≥ 4 mm) in systolic blood pressure in women with a greater waist-to-hip circumference ratio (WHR) and decreases in diastolic pressure in those who had used ART for a shorter period [21]. PA has also been shown to increase cardiorespiratory fitness [22], while high intensity exercise has been shown to increase peak volume of oxygen consumption (VO_2_peak) [23] among PWH. Resistance exercise has also been found to lead to increased lean mass percentage in PWH [17, 18]. Similarly, systematic reviews have also shown that PA is beneficial for body composition [24, 25], psychological conditions [26, 27], fitness and functional capacity [28, 29] and health-related quality of life (HRQOL) [29, 30] among PWH.

Although the pathways by which PA effects CMD indicators is not fully elucidated, two potential mechanistic pathways [31] have been proposed. First, PA may increase the anti-inflammatory cytokine adiponectin and second, it may decrease pro-inflammatory adipokines [32, 33]. Thus, it has been shown that in PWH, supervised endurance training decreases C-reactive protein, interleukin 6, interleukin 18 and tumor necrosis factor-alpha [34] and that supervised moderate intensity exercise is associated with improvement in C-reactive protein, plasma soluble CD14, d-dimer, interleukin 6 and interleukin 18 levels [35]. Physically active PWH have significantly lower advanced glycation end products, lower triglycerides, waist circumference, [36] and less lipodystrophy than non-active PWH [30]. In summary, PA is associated with both improved health indicators and mechanistic inflammatory pathways that are associated with future risk of CMD and all-cause mortality.

The benefits of PA for PWH, highlight the need to facilitate the implementation of PA for PWH, especially in places where public health systems face considerable resource-constraints. Despite the widely recognized health benefits of regular PA for PWH, studies have consistently shown that most PWH engage in insufficient PA [29, 30, 37–39]. In sub-Saharan Africa specifically, a region where nearly two thirds of the world’s population of PWH reside [40], representing a third of the 2.6 million disability life-years caused by cardiovascular disease among PWH globally [41], a large proportion of PWH do not engage in PA as part of their rehabilitation [42]. Furthermore, attrition rates from PA programs in intervention studies are approximately 30% [24, 43, 44]. Inadequate engagement in PA is believed to be due to the over-congested public health systems where most of the focus is directed toward delivering pharmacologic interventions to patients, with restricted time to offer other public health services [45]. Other barriers to CMD screening and management include low knowledge and perceptions of CMDs among PWH [46], poor education of health care providers in CMD management [47–49], staffing shortages [50, 51], and poor transportation and urban planning with limited safe green spaces for PA activities [52].

Because CMD is a leading and growing cause of morbidity and mortality among PWH, and because PA is strongly associated with improved CMD health outcomes in other populations, a thorough understanding of approaches to promote PA among PWH in sub-Saharan Africa will be a crucial first step to develop, responding to an unmet health need for this population. To positively change health behavior and promote PA in this population, there is a crucial need for public health leaders and health professionals to understand the elements and impacts of successful PA interventions [53]. Identifying the most effective components of PA interventions as well as their effectiveness will be prerequisites for synthesizing, implementing, and replicating successful interventions [54]. Unfortunately, there is a lack of consolidated data on PA and its impacts on health among PWH in sub-Saharan Africa, notably, the features of PA interventions that are successful and have been implemented effectively to improve health among PWH in the era of ART.

### Study aims

Our primary aim is to review the characteristics and efficacy of PA interventions to improve cardiometabolic outcomes, including body composition (e.g., waist circumference, body mass index, waist-to-hip ratio, total body weight), fitness and functional capacity, total cholesterol, lipids, triglycerides, glucose and blood pressure in PWH in sub-Saharan Africa. A secondary aim is to assess if these interventions also improve psychosocial outcomes, including HRQOL, anxiety, depression, self-efficacy, and social support.

#### Hypothesis

1. PA interventions are effective in improving cardiometabolic and psychosocial outcomes among PWH in sub-Saharan Africa.
2. Successful PA interventions are characterized by socioecological factors and the employment of behavioral change techniques such as motivation, goal setting, self-efficacy, social support, and feedback that are tailored for PWH in resource-poor settings.

## Methods

The review will follow the preferred reporting items for systematic review and meta-analysis protocols (PRISMA-P) guideline [55] of reporting systematic reviews. Additional file 1: Table S1 shows the populated PRISMA-P checklist for the review protocol. The protocol will be registered with PROSPERO and a detailed preregistered protocol will also be published.

### Inclusion criteria

The following criteria must be satisfied to be eligible for review:

1. Studies published between 2000 and the current date.
2. Population: includes adults (18 + years) living with HIV in sub-Saharan Africa.
3. Interventions: any PA-based intervention with a primary aim to improve cardiometabolic and psychosocial outcomes. This may or may not be explicitly stated in the study aim.
4. Comparator or control: No specified comparator or control but may include any passive (e.g., usual care) or active (e.g., alternative behavioral approaches) control group.
5. Outcomes: cardiometabolic, including but not limited to, body composition (including, waist circumference, body mass index (BMI), waist-to-hip-ratio, body weight), fitness and functional capacity, total cholesterol, triglycerides, lipids, glucose, blood pressure and psychosocial outcomes (including but not limited to, HRQOL, anxiety, depression, self-efficacy, and social support).
6. Study design: we will include randomized controlled trials and quasi-experimental study designs.

### Exclusion criteria

The following will be excluded from the review:

1. Manuscripts that do not include original data about the effects of PA on cardiometabolic and psychosocial outcomes.
2. Observational studies, case series, preprint, and theses will be excluded.

### Information sources

We will conduct a search in five reference databases, as follows: PubMed, African Index Medicus, Web of Science, Cumulative Index to Nursing and Allied Health Literature (CINAHL), and Embase. Articles published in any language will be included, being translated in Google translator when the language is not native to the authors. Reference lists of previous reviews and included papers will be searched to check for any further studies. The PubMed draft search strategy is presented in Additional file 2.

### Selection of sources of evidence

The review will be conducted in four phases: (i) searching for relevant articles using the search strategy, (ii) including or excluding articles based on their titles or abstracts, (iii) checking for article relevance in full-text articles, and (iv) looking for additional relevant articles by reviewing the reference lists of included articles and published relevant reviews.

#### Brief summary of data synthesis plan

### Screening

The full search output will be imported into Covidence software (Veritas Health Innovation 2020) for screening. This software will also assist in managing the study records and data throughout the review. Two authors (S.Z.M and J.M-G) will screen the first 250 abstracts in duplicate, in sets of 50. When agreement between the 2 authors exceeds Cohen κ > 0.90 for 2 successive sets of 50 abstracts, a single reviewer (S.Z.M.) will screen the remaining abstracts.

### Data extraction

Each selected study will be analyzed using a standardized consensual form adopted from Howlett et al. [53], to obtain the following data: characteristics of the sample, study design, study setting, intervention description, behavioral theories, PA measures used and results.

Specifically, we will extract the author’s name, year of publication, aim, inclusion and exclusion criteria for participants, characteristics of included participants (i.e., age, gender, stage of disease, comorbidity, and whether they were on ART).The following intervention details will also be recorded (name of the intervention (where applicable), exercise modality (i.e., aerobic, resistance or multimodal exercise), frequency and total duration of the intervention, setting, mode of delivery, theoretical framework informing the intervention, components of the intervention, and the summary of the findings), the outcomes of interest and their values at baseline and study completion, number of participants at baseline and study completion (including number of withdrawals), and the effectiveness and potential weaknesses of each intervention. In terms of PA outcome, the method of measuring PA, the number of participants randomly assigned and assessed, as well as the mean PA level at baseline and at post-intervention will be extracted. Missing data will be recorded as “missing in article”. Table 1 shows a summary of intervention content to be extracted.

**Table 1:** Data extraction table (adopted from Howlett et al. [53])

Extraction categories	Extraction items
General	Author(s); article title; type of publication (e.g., published article); country of origin
Methods	Design: aims/objectives of the study; target behaviour/s; study design (including control groups); inclusion and exclusion criteria; recruitment and sampling methods (including unit of randomization and blinding); unit of allocation; power calculations
	Participants: population type; inclusion and exclusion criteria; number of participants; age; gender; weight status; CD4 count; ART; ethnicity
Intervention features	General description of the intervention; frequency and length of sessions; intervention duration; intervention setting; intervention provider; delivery format; behaviour change techniques; theoretical basis; Mean health-related outcome measure at baseline and at post-intervention; potential weakness of the intervention
Outcomes	Primary outcome (cardiometabolic outcomes): method and unit of measurement; type of measurement (e.g., subjective); follow-up duration and frequency; mean and standard deviation at baseline, post intervention, and follow-up; effectiveness at post intervention and follow-up; effect size; attrition rate
	Secondary outcomes (psychosocial outcomes): method and unit of measurement; type of measurement (e.g., subjective); follow-up duration and frequency; mean and standard deviation at baseline, post intervention, and follow-up; effectiveness at post intervention and follow-up; effect size; attrition rate

### Methodological quality of the included studies

An appropriate scale based on the study design of the article will be used to assess the methodological quality of the included studies. Specifically, the revised tool to assess risk of bias in randomized trials (RoB 2) [56] will be used to assess the methodological quality of the included randomized controlled trial studies. In terms of quasi-experimental study designs, methodological quality will be assessed using the relevant items from the Downs and Black [57] checklist. Using the 10-item criteria, studies will be given a positive (greater than 50% criteria met), neutral (50% criteria met), or negative (less than 50% criteria met) rating. Two persons (SZM and JM-G) will be assigned to independently review the quality of the studies. Where the reviewers do not agree, a third reviewer will be consulted (MS or MH) and the final decision regarding the rating of the study will be resolved by joint discussion and consensus among the reviewers as a group.

### Certainty evidence

Certainty of evidence will be evaluated using the Grading of Recommendations Assessment, Development and Evaluation guidelines (GRADE) [58]. We will use the GRADE evaluation process to develop “Evidence Profiles” tables of the outcomes of interest in each included study based on full-text narrative abstraction and methodological quality scoring. The GRADE criteria will entail assessing the following five domains: risk of bias, inconsistency, imprecision, indirectedness, and publication bias. Certainty relating to the extent to which the body of evidence represents a true estimate of effect for each outcome will be deemed to be high, moderate, low, or very low in accordance with the GRADE criteria [58]. Table 2 shows the GRADE criteria. Studies start as high quality evidence but can be downgraded for serious flaws that violate the five domains of the GRADE criteria. For each GRADE table, we will provide an estimate of certainty or the strength of evidence (from high to very low) and a direction of effect (positive, no effect, or negative). However, we will only provide a GRADE for studies that score ≥ 50% on methodological quality rating. SZM and JM-G will be assigned to independently review the certainty of evidence. Where the reviewers do not agree, a third reviewer will be consulted (MS or MH) and the final decision regarding the rating of the study will be resolved by joint discussion and consensus among the reviewers as a group.

**Table 2:** The GRADE criteria

High	We are very confident that the true effect lies close to that of the estimate of the effect
Moderate	We are moderately confident in the effect estimate: the true effect is likely to be close to the estimate of the effect, but there is a possibility that it is substantially different
Low	Our confidence in the effect estimate is limited: the true effect may be substantially different from the estimate of the effect
Very low	We have very little confidence in the effect estimate: the true effect is likely to be substantially different from the estimate of effect

### Synthesis and analysis of results

The emanating results found using the outlined methodology will provide a summary of the current research and an assessment of the quality of individual studies. Results will be reported in the form of a narrative synthesis summarizing data on the effectiveness and the characteristics/components of effective interventions to improve cardiometabolic and psychosocial outcomes among PWH in sub-Saharan Africa. The narrative will take the methodological quality of the studies and certainty of evidence into perspective. The review will also highlight the strengths and weaknesses of PA interventions amongst PWH in sub–Saharan Africa. Based on the evidence, recommendations will be made to improve and further strengthen the interventions.

Meta-analyses will be conducted if sufficient studies assess the same outcome (e.g., quality of life or change in weight), compare similar groups (e.g., standard of care versus a well-described PA intervention) and the articles provide the mean and standard deviation of the outcome variables immediately after the end of the intervention to evaluate the immediate between-group effects [59]. Meta-analyses will be conducted with Stata software version 17.0 (StataCorp LP, College Station, TX) computer software and the random-effects model for outcomes will be used. We will calculate the weighted mean difference (WMD) and 95% confidence intervals for the means for continuous outcomes whenever possible. On the other hand, for dichotomous outcomes, the odds ratio, absolute difference in odds, relative risk (RR), risk difference (RD), and the number needed to treat (NNT), and 95% confidence intervals will be calculated whenever possible. A *p* value of less than 0.05 will show statistical significance for overall effect of the intervention. For heterogeneity between studies (*I*^2^), a *p* value of less than 0.1 will be considered as statistically significant [60]. We will consider an *I*^2^ ≤ 40 as low heterogeneity, *I*^2^ > 40–75% as moderate and *I*^2^ > 75% as a high heterogeneity [61]. Subgroup analyses will be performed whenever possible to evaluate if PA interventions are associated with differences among the following pre-specified sub-groups: (1) female and male gender, (2) BMI > 30 (i.e., traditionally defined obesity) versus BMI < 30, (3) blood pressure: systolic > 120 mm Hg, diastolic > 80 mm Hg versus systolic < 120 mm Hg, diastolic < 80 mm Hg,(4) HbA1c < 5.7% versus > 5.7% and PA of < 150 min/wk versus > 150 min/week, (5) ART vs no ART at enrolment.

## Discussion

This systematic review will identify and synthesize the current evidence regarding the effectiveness and characteristics of PA-based interventions to improve cardiometabolic and psychosocial outcomes among PWH in sub-Saharan Africa. We also hope to identify and highlight the strengths and weaknesses of these interventions. Additionally, based on the evidence, recommendations will be made on possible strategies to adopt when designing PA interventions for PWH in sub-Saharan Africa to improve and strengthen the selection of these interventions for recommendation, further study and/or implementation in public health programs.

It is important to assess the effectiveness and characteristics of PA interventions for PWH in sub-Saharan Africa because, regardless of the observed benefits, most PWH in sub-Saharan Africa are still not engaging in adequate PA as part of their health promotion activities [42] and withdrawal rates from PA programs in sub-Saharan Africa studies are up to 30% [23, 43, 44]. We focus on PWH in sub-Saharan Africa not only because of the high prevalence of HIV in the region, but also because PWH in sub-Saharan Africa face unique societal and health system challenges compared to other regions of the world and have particularly high rates of obesity with modern ART regimens [11, 12, 16, 62].

This review will aid researchers in designing effective and contextualized PA interventions for PWH in sub-Saharan Africa. We posit that an understanding of the effectiveness and characteristics of PA interventions for PWH in sub-Saharan Africa will assist in the design and implementation of context-sensitive interventions that are more suitable in the sub-Saharan African environment, and specifically for PWH. The recommendations emanating from this systematic review should serve well the needs of researchers, policy-makers, and other public health decision-makers in influencing the future agenda for efforts to explore and promote PA among PWH in sub-Saharan Africa.

## Supplementary Information

**Additional file 1. **PRISMA-P 2015 Checklist.

**Additional file 2. **Search strategy for PubMed.

## Data Availability

Not applicable.

## Abbreviations

ART: Antiretroviral therapy
CMD: Cardiometabolic disease
HIV: Human immunodeficiency virus
HbA1c: Hemoglobin A1C
HRQOL: Health-related quality of life
LS7: Life’s Simple 7
PA: Physical activity; PWH: People living with HIV
