# Supplementary material for: Characteristics and efficacy of physical activity interventions to improve cardiometabolic and psychosocial outcomes in people living with HIV in sub-Saharan Africa: a protocol for a systematic review
Source: Syst Rev. 2023 Feb 23;12:24. doi: 10.1186/s13643-023-02186-5 (PMC9948386; doi:10.1186/s13643-023-02186-5)
Supplement: Supplementary file 2 — Additional file 2. Search strategy for PubMed. [file 13643_2023_2186_MOESM2_ESM.docx]

**Search strategy for PubMed**

Physical activity

((("Exercise"[Mesh] OR "Sports"[Mesh] OR "Physical Fitness"[Mesh] OR "Cardiorespiratory Fitness"[Mesh] OR "Movement"[Mesh] OR interval[tiab] OR lifestyle*[tiab] OR physical activit*[tiab] OR endurance[tiab] OR weight-lifting[tiab] weight-bearing[tiab] OR high-intensity[tiab] OR HIIT[tiab] OR cardiorespiratory[tiab] OR cardio[tiab] OR resistance[tiab] OR strength[tiab] OR athletic*[tiab] OR working-out[tiab] OR work-out[tiab] OR strength-training[tiab] OR sport*[tiab] OR team[tiab] OR steps[tiab] or pedometer[tiab] OR aerobic*[tiab] OR muscle-strengthening[tiab] OR bone-strengthening[tiab] OR stretching[tiab] OR exercise*[tiab]) AND (*Active[tiab] OR activit*[tiab] OR fitness[tiab] OR training[tiab] OR exercis*[tiab] OR lifting[tiab] OR conditioning[tiab] OR intervention*[tiab] OR physical[tiab] OR physically[tiab] OR* exercise*[tiab] OR physical activit*[tiab]))

HIV

((Human[tiab] AND Immunodeficiency[tiab] AND Virus[tiab]) OR (Acquired[tiab] AND Immune[tiab] AND Deficiency[tiab] AND Syndrome[tiab]) OR “Human Immunodeficiency Virus” OR “Acquired Immune Deficiency Syndrome Virus” OR AIDS[tiab] OR AIDS Virus[tiab] OR Seronegativities[tiab] OR Seronegativity[tiab] OR HIV[tiab] OR hiv-1[tiab] OR hiv-2[tiab] OR hiv1[tiab] OR hiv2[tiab] OR hiv infect*[tiab] OR “human immune deficiency virus”[tiab] OR “human immuno-deficiency virus”[tiab] OR “human immune-deficiency virus”[tiab] OR “acquired immuno-deficiency syndrome”[tiab] OR “acquired immune-deficiency syndrome”[tiab] OR HIV/AIDS[tiab] OR HIV-positive[tiab] OR HIV[ti] OR HIV/AIDS[ti] OR "HIV"[Mesh] OR "HIV Long-Term Survivors"[Mesh] OR "HIV Seronegativity"[Mesh] OR "HIV Infections"[Mesh] OR "HIV Seroprevalence"[Mesh] OR "HIV-2"[Mesh] OR "HIV-1"[Mesh] OR "HIV Seropositivity"[Mesh] OR HIV[tiab]))

Sub-Saharan Africa

("Africa"[Mesh] OR "Africa South of the Sahara"[Mesh] OR Africa*[tiab] OR Subsaharan[tiab] OR sub-saharan[tiab] OR Cameroon[tiab] OR Central African Republic[tiab] OR Chad[tiab] OR Congo[tiab] OR Democratic Republic of the Congo[tiab] OR Equatorial Guinea[tiab] OR Gabon[tiab] OR “Sao Tome and Principe” OR Burundi[tiab] OR Djibouti[tiab] OR Eritrea[tiab] OR Ethiopia[tiab] OR Kenya[tiab] OR Rwanda[tiab] OR Somalia[tiab] OR South Sudan[tiab] OR Sudan[tiab] OR Tanzania[tiab] OR Uganda[tiab] OR Angola[tiab] OR Botswana[tiab] OR Eswatini[tiab] OR Lesotho[tiab] OR Malawi[tiab] OR Mozambique[tiab] OR Namibia[tiab] OR South Africa[tiab] OR Zambia[tiab] OR Zimbabwe[tiab] OR Benin[tiab] OR Burkina Faso[tiab] OR Cabo Verde[tiab] OR “Cote d'Ivoire” OR Gambia[tiab] OR Ghana[tiab] OR Guinea[tiab] OR Guinea-Bissau[tiab] OR Liberia[tiab] OR Mali[tiab] OR Mauritania[tiab] OR Niger[tiab] OR Nigeria[tiab] OR Senegal[tiab] OR Sierra Leone[tiab] OR Togo[tiab] OR Xhosa[tiab] OR Xosa[tiab] OR Bantu[tiab] OR Rundi[tiab] OR Rwanda[tiab] OR Shona[tiab] OR Zulu[tiab] OR Lingala[tiab] OR Swahili[tiab] OR Hausa[tiab] OR Yoruba[tiab] OR Ibo[tiab] OR Igbo[tiab] OR Kongo[tiab] OR Fulani[tiab] OR Amharic[tiab] OR Oromo[tiab] OR Chewa[tiab] OR Sotho[tiab] OR Showa[tiab] OR Khoisan[tiab] OR  KhoiSan[tiab] OR  Khoi-San[tiab] OR *Khoe-San[tiab] OR Malagasy[tiab] OR Afrikaans[tiab] OR Hausa[tiab] OR Songhay[tiab] OR Songhai[tiab] OR Berber[tiab]*))

Clinical trial

("clinical trial"[pt] OR "random allocation"[Mesh] OR random*[tiab] OR clinical trial[tiab] OR clinical study[tiab] OR controlled trial[tiab] OR controlled study[tiab] OR "Pilot Projects"[Mesh] OR ((phase i*[tiab] OR phase 1*[tiab] OR phase 2*[tiab] OR phase 3*[tiab]) AND (study[tiab] OR studies[tiab] OR trial[tiab])) OR program*[tiab] OR intervention[tiab] OR pilot*[tiab] OR strateg*[tiab])
